# Preventing Revictimization Through a Web-Based Intervention for Primary Caregivers of Youth in Care (EMPOWERYOU): Protocol for a Randomized Factorial Trial

**DOI:** 10.2196/38183

**Published:** 2022-10-24

**Authors:** Nina Heinrichs, Antonia Brühl

**Affiliations:** 1 Department of Psychology, Clinical Psychology and Psychotherapy University of Bremen Bremen Germany

**Keywords:** multiphase optimization strategy, foster children, parenting, revictimization, web-based intervention, online intervention

## Abstract

**Background:**

Children in foster care are at a higher risk for relationship problems than their counterparts raised by their biological family because of higher exposure to or prevalence of neglect or maltreatment early in life. Consequently, these children may also show more challenging behavior in their foster families, which in turn increases the parental stress experience of foster caregivers. Furthermore, the children may engage in a vicious cycle of risky relationship behaviors and expectations that put them at a greater risk for revictimization.

**Objective:**

To support foster caregivers in reducing the risk for revictimization, several intervention modules delivered via the internet were developed using a consumer-based approach (phase 1 of the multiphase optimization strategy). This project (phase 2 of the multiphase optimization strategy) aimed to develop a sustainable intervention by selecting promising intervention components based on their contribution to the outcome.

**Methods:**

In a 2^4^ factorial trial, a total of 317 foster caregivers with children aged 8 to 13 years are randomly assigned to 1 of 16 conditions. The primary outcome is the rate of revictimization from baseline to 3 months after intervention. Secondary outcomes include risk-taking and functional behaviors in relationships. All caregivers will receive access to all the intervention components after the follow-up assessment. The participants assigned to the condition with all component levels *on* are expected to show the best improvement in the primary and secondary outcomes.

**Results:**

Recruitment and data collection for the factorial trial started in March 2022 and is ongoing. As of October 2022, we recruited 181 families. Although it is difficult to predict the exact study timeline owing to COVID-19 pandemic–related delays, results are expected in February 2024.

**Conclusions:**

There is a need for easily accessible information related to raising children in foster care who have experienced early life adversities to interrupt the cycle of violence and enhance the developmental pathway of health and emotional stability. It might be useful, in addition to generally useful parenting information (eg, parental self-care or emotion regulation management), to specifically focus on the needs of these caregivers (eg, how to support the child to reduce dysfunctional relationship behaviors that may have developed because of early adverse experiences).

**Trial Registration:**

ClinicalTrials.gov NCT05235659; https://clinicaltrials.gov/ct2/show/NCT05235659

**International Registered Report Identifier (IRRID):**

DERR1-10.2196/38183

## Introduction

### Background

Exposure to potentially traumatic events and being bullied by peers or siblings in childhood constitute forms of victimization or revictimization, which may be associated with severe long-term effects on mental health [1-3], including anxiety, depression, or suicidality [4]. We include the experience of different forms of maltreatment (emotional, physical, and sexual abuse; neglect; and intimate partner violence) as well as bullying experiences in the definition of revictimization in this trial.

Given that childhood victimization leads to an increased vulnerability for subsequent revictimization in adolescence [5,6], findings highlight a strong need for evidence-based prevention programs targeting children with a history of maltreatment or bullying as a high-risk population for revictimization. Although all types of maltreatment in childhood were found to be associated with revictimization [7] and mental health, one study with adolescent girls in child welfare found that the emotional type of maltreatment showed the strongest link to revictimization in a cross-sectional study using self-report of the types of child maltreatment experienced [8]. However, when using a population sample, sexual maltreatment increased the risk for revictimization the most [7]. In a recent meta-analysis, Scoglio et al [9] identified the following risk factors in most studies on the association between sexual victimization and revictimization: risky sexual behavior, further maltreatment experiences in childhood, presence of posttraumatic stress disorder (PTSD), and emotional dysregulation. In contrast, protective factors have rarely been examined. Only parental caregiving was identified as a protective factor. Unfortunately, many of the included studies were cross-sectional, and it is not always clear which key mechanism is driving the link (eg, why the presence of PTSD is increasing risk).

### Children in Foster Care as an Example of a High-Risk Group for Revictimization

Children are often placed in foster care because of early adverse experiences in their family of origin, including maltreatment, with the majority experiencing neglect and emotional maltreatment, followed by physical and sexual abuse [10]. These children usually show comprehensive problems in relationships, including foster parent–child relationship, relationship with siblings [11], and peer relationships [12]. Furthermore, many children in foster care are affected by PTSD or attachment disorders [13] and show externalizing problem behaviors, which increase parental distress in (foster) parents [14]. The consequences of maltreatment (eg, PTSD-related symptoms and cognitions, such as negative self-appraisal; maladaptive cognitions of others and the world, eg, concerning the reliability and trustworthiness of others; and threat of harm) have been discussed to be causally involved in the risk for revictimization, although direct mediating effects were only established for threat of harm [15].

### Interventions to Support Foster Caregivers

Many parenting programs are designed to equip (foster) parents with strategies for increasing positive behaviors in their children and to support them in appropriately managing externalizing problem behavior [16]. Although parenting programs, in general, are a very promising approach to changing child behavior (via changed parenting behavior and reduced parental stress [17]), it has rarely been investigated whether parents may also help to lower the risk for revictimization in children with high risk due to adverse (early) childhood experiences. Warm and responsive parenting is associated with protective effects on children’s resilience to victimization [18], indicating that such parenting programs may be beneficial for coping with victimization and revictimization experiences. However, it is unclear whether parents could also be equipped with the knowledge and skills required to empower children with high risk for revictimization and thereby lower the risk for revictimizing experiences. Burke et al [19] outlined that *parental support* did not change the occurrence of victimization. Some authors also pointed out that some intervention components may be less effective than others in preventing or reducing child maltreatment experiences. Gubbels et al [20] concluded in their review and meta-analysis that “improving parental personal skills, improving problem-solving skills, and stimulating children’s prosocial behavior should not be the main focus of parental training programs for preventing and reducing child maltreatment” [20]. However, many of these have been identified as promising components to successfully change child externalizing behavior [21], suggesting that the 2 different outcomes may be the result of different pathways of change. Child externalizing problem behavior could be driven by engines different from the risk for revictimization. The intervention model theory of change is key to determining the best intervention components that are most likely to cause changes in the preferred outcome domain [22]. Furthermore, there is some evidence that also challenges the impact (foster) parents may have on the developmental adaptation of their children in care, and the dynamic and reciprocal processes between children and parents that build the foundation for many social learning–based parenting approaches have not been fully supported in a sample of children in foster care in the Netherlands [14]. Although the children’s behaviors affected the distress levels of the foster caregivers, the foster caregivers’ stress did not affect the children’s behaviors. Although this research group discussed a number of potential reasons for the lack of support for a transactional model (eg, foster parents may be expressing their distress less than biological parents, they could potentially give the child away, or children are less vulnerable to parental distress because they are accustomed to worse), this study may indicate that foster parent–child interactions may differ when children were maltreated in the past or at least the focus of the intervention may need to be shifted. For example, Burke et al [19] suggested teaching parents to “be more responsive and connected to their children when they are experiencing difficulties” [19] instead of equipping them with parent management skills more generally.

In sum, few evidence-based parenting programs for foster parents are available, and most of them include a package of intervention strategies [20] and require comprehensive training and parental participation (eg, in-home training as in Attachment and Biobehavioral Catch-up or Keeping Foster Parents Trained and Supported [23,24]). However, (foster) parent participation is challenging [25]. Furthermore, the intervention model and key drivers of change are not specifically tailored to the factors that put children with maltreatment experiences at a higher risk for future revictimization [26]. There is a clear need to identify behaviors and pathways that are responsible for revictimization [27] in this population.

### This Study

We use the multiphase optimization strategy (MOST) framework [22] to prepare and optimize an intervention for foster parents. The intervention components to be tested in phase 2 of MOST (this factorial study) were developed based on the phase 1 results. Multimedia Appendix 1 [27-32] provides a brief summary of the phase 1 results.

**Figure 1:**
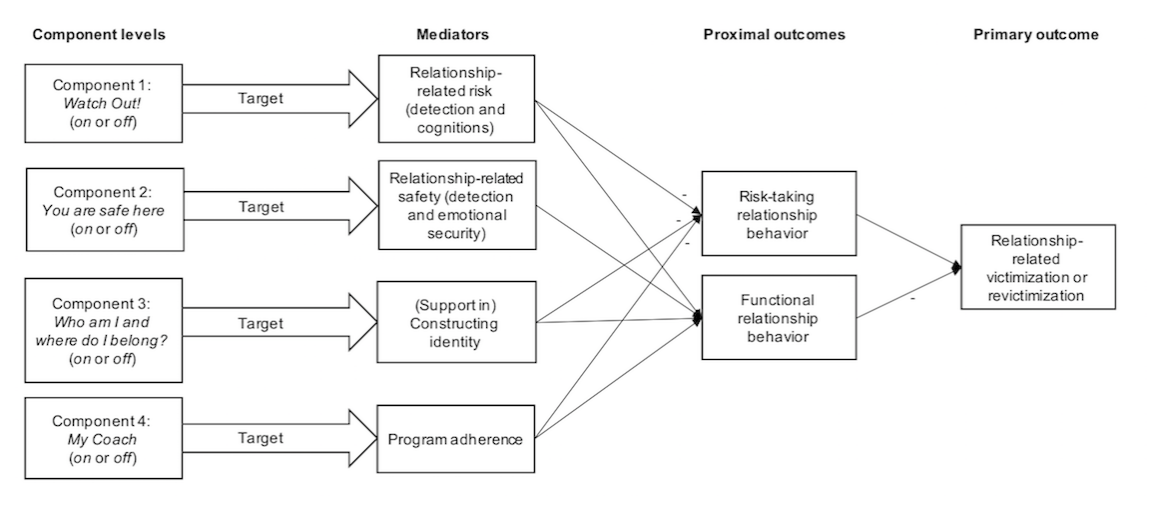
Conceptual model of the EMPOWERYOU intervention for primary caregivers of youth in care.

We built our conceptual model (Figure 1) and selected the following domains as targets for the intervention (*mediators* in the conceptual model):

1. Relationship-to-harm beliefs, which emphasize the degree to which a child believes that close relationships include harm
2. The threshold for risk detection, which, if lowered, leads to a delayed notice and, consequently, delayed response to danger cues in relationships

We combine these domains into 1 mediator called *relationship-related risk.*

1. A lack of relationship skills to build up and maintain positive and safe relationships
2. Difficulties to detect safety signals and feeling emotionally secure in close relationships

We combine these domains into one mediator called *relationship-related safety.*

1. The emotional significance of a child’s origins and the child’s current foster family for *constructing a coherent identity*. This domain includes the recognition and sensitive responding of caregivers to the emotional significance of both families for the child’s identity development.

We developed promising intervention components and specified how we expect these to change the risks of revictimization experiences and their consequences.

### Objectives and Hypotheses (for Phase 2 of MOST)

The primary aim of this study is to examine the effects of each candidate component developed based on the conceptual model and delivered via an internet-based prevention program to the primary caregivers of preadolescent youth in care on revictimization in the form of conventional crime, child maltreatment, peer and sibling victimization, sexual victimization, witnessing and indirect victimization, and cyberbullying. The ultimate goal is to choose the candidates that best reduce and prevent revictimization from a set of *4 components* with *2 levels each (on/off)*. The primary and secondary research objectives are presented in Textbox 1.

We will examine the hypotheses specified in Textbox 2, which are related to the *main effects of the intervention components* (3 content components and 1 adherence component).

**Primary and secondary research objectives.**

**Primary research** **objectives**

1. To examine the efficacy of the selected candidate components on the primary outcome, the risk of revictimization, at follow-up (ie, 3 months after intervention; approximately 24 weeks after baseline)
2. To examine the efficacy of the selected candidate components on the secondary outcomes (risk-taking behavior and functional behavior in relationships with the caregiver, siblings, peers, and others; Multimedia Appendix 2 [33-55] provides a full list of outcomes [Heinrichs, N, unpublished data, April 2021; Niestroj, S, unpublished data, September 2021; Zemp, M, unpublished data, 2011; Brühl, A, unpublished data, April 2021; Heinrichs, N, unpublished data, August 2020; Heinrichs, N, unpublished data, March 2021]) at posttest assessment (ie, 1 week after intervention; approximately 12 weeks after baseline)

**Secondary research objectives**

1. To test the enduring effects of the selected components on the secondary outcomes at 3-month follow-up
2. To test the mediating effects of theory-driven factors (Multimedia Appendix 2 provides a full list of potential mediators) on the relationship between the selected components and the secondary outcomes
3. To explore whether there are any interaction effects between components on the primary or secondary outcomes
4. To conduct exploratory analyses of potential moderators

**Hypotheses related to the main effects of the intervention components.**

**Hypotheses to be examined**

1. Component *Watch Out!* deals with relationship-related risks, and we hypothesize that receiving this component will result in a better detection of risk signals in relationships and less risk-taking cognitions. This will lead to less relationship-related risk-taking behavior, which, in turn, will result in reductions in revictimization experiences and other secondary outcomes.
2. Component *You are safe here* deals with relationship-related safety, and we hypothesize that receiving this component will result in a better detection of relationship-related safety signals in relationships and more emotional security. This will lead to more functional relationship behavior, which, in turn, will result in reductions in revictimization experiences and other secondary outcomes.
3. Component *Who am I and where do I belong?* deals with the construction of identity, and we hypothesize that receiving this component will increase parental support for the child’s efforts in constructing a coherent identity. This will lead to less risk-taking behavior and more functional relationship behavior, which, in turn, will result in reductions in revictimization experiences and other secondary outcomes.
4. Component *My Coach* deals with professional support, and we hypothesize that professional support from a parent coach who is facilitating each component will result in higher program adherence or engagement, which will yield larger intervention effects on the primary and secondary outcomes than without professional support.
5. We hypothesize that there will be an interaction effect between the relationship risk and relationship safety components. When both components are present, the effect will be larger compared with when only one of them is present.
6. We hypothesize that there will be an interaction effect between each component and the professional support component such that with professional support, the effects of each component will be larger than those without professional support via greater adherence of the caregiver.

We will also answer the following four questions to establish how well the conceptual model captures the relevant mechanisms of the intervention (mediation analyses, adapted from the study by Smith et al [56]): how well will the three mediators (relationship-related risk, relationship-related safety, and constructing an identity) predict the occurrence of revictimization, how well does each module content (*Watch Out*!, *You are safe here*, and *Who am I and where do I belong*?) and the coach evoke each of the 3 mediators, how much of the relationship between the intervention’s content components and revictimization is explained by the 3 hypothesized mediators, and how much variability between the content components and revictimization will remain unexplained.

Furthermore, the following variables were identified as potential moderators of intervention (component) efficacy based on the literature review in phase 1:

1. Child executive functioning (ie, an impaired “ability to shift, inhibit, and focus attention; maintain focus in the face of distracting information” [28]): Reduced executive functioning will lead to smaller intervention effects.
2. Gender of the child [27,57]: We expect worse outcomes in girls than boys.
3. Type of maltreatment [7-9]: Sexual maltreatment experiences will lower the intervention effect compared with other types.
4. Contact with the family of origin [58]: We assume that conflictual contact may reduce intervention effects.
5. Caregiver’s history of child maltreatment [59]: We expect caregivers with such a history to benefit less from the intervention.

Taken together, we use the MOST framework [22] to optimize a web-based program for foster parents, which comprises 4 intervention components. To optimize the program, the primary aim of this study is to examine the efficacy of each component on the primary and secondary outcomes. This paper outlines the protocol (version 01) or the factorial trial, following the SPIRIT (Standard Protocol Items: Recommendations for Interventional Trials) guidelines for clinical trials (Multimedia Appendix 3 provides the SPIRIT checklist).

## Methods

### Ethics Approval

This study is conducted in accordance with the Declaration of Helsinki. This study was approved on September 1, 2021, by the University of Bremen Ethics Committee (no. 2021-09). Any changes to this protocol will be submitted to the institutional review board for notification and approval.

### Study Design

The EMPOWERYOU project (funded by the German Ministry of Education and Research, Project code FKZ 01KR1806D) aims to develop and optimize a web-based parenting program by using the MOST framework [22]. This study will use a 2×2×2×2 full factorial design by randomly allocating participants to 1 of 16 experimental conditions (Table 1). To estimate the main effects of the 4 intervention components and their interactions, data from all the experimental conditions will be used. For example, the effect of the component *Relationship-related risk* will be estimated by comparing the mean of the experimental conditions 1 to 8 with the mean of the conditions 9 to 16 (Table 1). Families in the first condition will not receive any experimental component but will be delivered the 2 basic intervention modules that every caregiver receives. Families will be informed that everyone may access all modules and that the sequence and timing of each module will be based on a randomization procedure.

**Table 1:** Experimental intervention conditions (optimization schema).

Experimental condition	Component 1: *Watch Out*!—relationship-related risk	Component 2: *You are safe here*—relationship-related safety	Component 3: *Who am I and where do I belong?*—identity	Component 4: *My Coach*—professional support
1	Off^a^	Off	Off	Off
2	Off	Off	Off	On^b^
3	Off	Off	On	Off
4	Off	Off	On	On
5	Off	On	Off	Off
6	Off	On	Off	On
7	Off	On	On	Off
8	Off	On	On	On
9	On	Off	Off	Off
10	On	Off	Off	On
11	On	Off	On	Off
12	On	Off	On	On
13	On	On	Off	Off
14	On	On	Off	On
15	On	On	On	Off
16	On	On	On	On

^a^Not included in the intervention.

^b^Included in the intervention.

### Sample Size Calculations

Anticipated effect sizes were estimated from the reported effect sizes for victimization and revictimization interventions, which also included (1) facilitating risk detection skills; (2) social skills, such as problem-solving or conflict management; (3) skills to build healthy relationships; and (4) the ability to reflect on own expectations that relationships will include harm [28,60,61]. We expect small to moderate effect sizes between Cohen *d*=0.28 and Cohen *d*=0.46 for the main effects of specific intervention components (component 3: 0.46, component 4: 0.28, and component 6: 0.32). Thus, a sample size of 317 was determined as necessary to detect the smallest anticipated effect size of Cohen *d*=0.28. To detect this difference with the analysis of covariance as the suggested method for component selection by the developer of the factorial design (groups=2, *df*=1, covariate=1) with 80% power at α=.10 per intervention component or interaction, a sample size of 317 is required (calculated with FactorialPowerPlan SAS Macro provided by Dr Collins [62]). We will use an intention-to-treat approach, although only one family dropped out during the intervention period in the pilot study. In contrast, findings from the pilot study showed that 26% (4/15) of the recruited caregivers that registered for the website dropped out before the intervention. Therefore, we consider a dropout rate (before the allocation to condition) of 26%. Therefore, we aim to recruit a total of 429 families. Recruitment will be stopped as soon as we reach the sample size needed for the analysis (N=317).

### Procedure

#### Inclusion and Exclusion Criteria

We will only allow primary caregivers (foster or adoptive) of youth in care aged 8 to 13 years. Families indicating acute child endangerment during the web-based screening assessment (using 5 self-developed items) will be excluded. We will further exclude caregivers with an insufficient knowledge of German language, short-term foster families (“Bereitschaftspflege”), or kinship care (“Verwandtenpflege”). Excluded families will be provided with professional advice and referrals for services if desired.

#### Recruitment

We will recruit via a national association for foster and adoptive parents (Bundesverband der Pflege- und Adoptivfamilien), regional youth welfare institutions, and self-help organizations in Germany. Furthermore, we have active social media campaigns running to support recruitment.

#### Randomization and Blinding

Families will be randomized to 1 of the 16 experimental conditions via a database that uses concealed, computer-generated [63], permuted block randomization, with stratification by child’s gender and with fixed block sizes of 16 (conditions were randomized within each block). Randomization procedures will be completed by another research group within the EMPOWERYOU consortium that is not associated with the intervention trial otherwise (Neuropsychology at the University Hospital Aachen, Germany) to minimize the occurrence of potential biases (eg, biases that may arise through primary caregivers’ or researchers’ preferences). Study staff at the University of Bremen will not view the allocation sequence to minimize researchers’ prediction biases. The database will not reveal participants’ treatment conditions to the study staff until after the family’s eligibility is verified (after preintervention assessment). Families will be informed of their allocation status after baseline data collection is completed to ensure that participants are blind to allocation during the initial assessment. In the case of instances of harm or severe abuse to a child being reported by a participant, the allocation status of the participant will be unblinded. All cases of unblinding will be reported to the Data Safety and Monitoring Board (DSMB).

### Informed Consent

Informed consent will be obtained from each participant (caregiver and child) on the web. Interested study participants can register on the website and then receive the information sheet and consent form on the web. The caregivers are informed about the objectives, study procedure, the rights and obligations of all those involved, and the procedure and data processing using the information and consent forms. If the participants have any questions about the documents or the study, they can call the study team at any time. The phone number will be clearly indicated on the website. Each caregiver is asked to provide active opt-in consent for their own participation and the participation of their child in care. Multimedia Appendix 4 provides the informed consent form in German language.

### Intervention

#### Intervention Accessibility and Orientation

**Figure 2:**
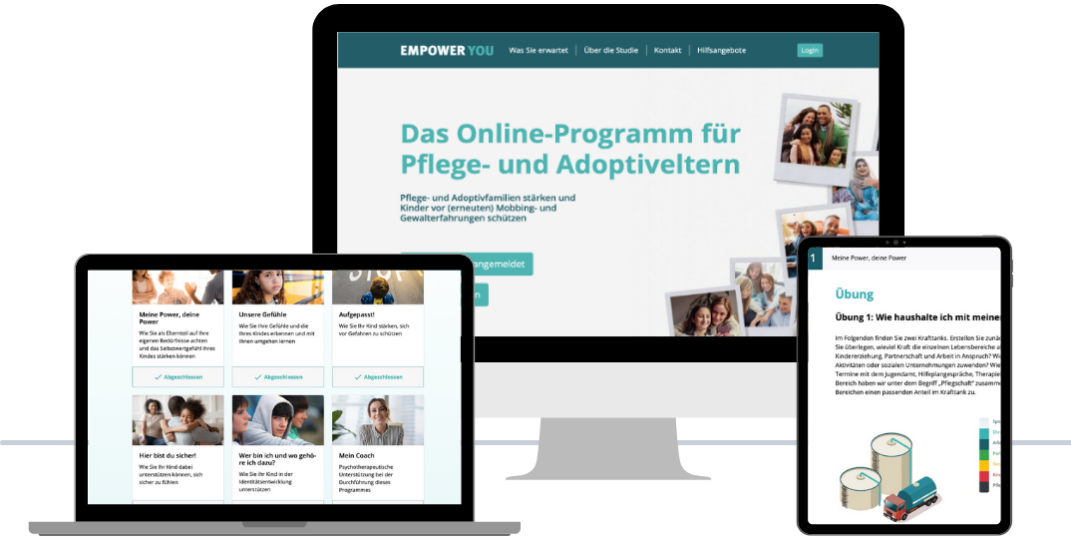
Website of the EMPOWERYOU program for caregivers.

Caregivers will use a password to access the program (Figure 2). All parents will be offered to participate in a “welcome call” with the coach before starting with the first module. During the phone call, the procedure of the intervention and the adverse event assessment will be explained.

#### Duration to Complete Each Module

During the program, parents will have 2 weeks to complete 1 module, with each module taking about 90 minutes to work through (including “homework” in the form of exercises with the child).

#### Duration of the Intervention Package

Caregivers can work through the web-based program for a maximum of 10 weeks (conditions with the 3 candidate components *on* resulting in 6 weeks plus 4 weeks for the 2 basic modules). The duration of the intervention is reduced by up to 4 weeks if caregivers receive the basic modules only (experimental condition 1; Table 1).

#### Content of Each Module

We have 6 intervention modules out of which only 4 will be put to test because modules 1 and 2 are already well investigated, with promising mental health benefits for the participants besides revictimization outcomes [20,21,64,65]. In addition to the 2 “basic modules,” a website area with literature recommendations and information texts on child maltreatment, self-injury, and suicidal behavior allows us to offer some level of support across all conditions, including the condition with the lowest component level across all intervention modules. The 6 intervention modules are described in detail in Textbox 3.

The 6 modules are embedded in a common website and independent of family assessment. The assessment is conducted via a professional tool for web-based surveys (refer to the *Data Collection for the Primary and Secondary Outcomes* section). The web-based intervention includes various multimedia features (texts, slides, videos, fictional audio recordings, and interactive tasks) to make the program easily accessible and attractive.

**The intervention modules.**

**Module *My power, your power*—facilitating parental self-care and the self-worth of the child**

- Caregivers learn how to recognize their own needs and how to implement resources-enhancing strategies in everyday life. Caregivers are encouraged and guided on how to facilitate their partnership quality (as an important resource for the caregiver and the child) and how to promote the child’s self-worth.

**Module *Our feelings*—facilitating the emotional regulation of the child and the caregiver**

- The second module provides basic knowledge about emotions and their functions. Caregivers are supported in recognizing and communicating their emotions as well as the child’s emotions. Parents are encouraged to attend to their child’s emotional needs, reflect on their emotions, and learn to keep them separate from their own emotional state, or at least recognize the difference and attempt to understand their child’s emotional reactions (eg, through storytelling and behavior attribution exercises). Emotional regulation strategies for the parent and the child are introduced and practiced.

**Module *Watch Out!*—improving relationship-related risk detection and the self-protective behavior of the child**

- Parents gain access to information on how they can support their child in noticing and processing risk signals (eg, combining risk signals). Exercises with the child that provide access to knowledge about children’s rights, inhibit risk-taking behavior, and enhance self-protective behavior are included. Examples on how to talk (with the child) about victimization (eg, bullying experiences) are provided, and a brief section on media literacy completes this module.

**Module *You are safe here*—improving relationship-related safety and facilitating solid relationships with family and peers**

- This module includes knowledge about attachment and emotional security, and parents are supported in enhancing the child’s feeling of safety in close relationships. Parents are encouraged to reflect on their own biography and that of their children to recognize functional or dysfunctional cognitions and assumptions about close relationships that may shape the way relationships are perceived or behaved in today.

**Module *Who am I and where do I belong?*—supporting the child in constructing an identity**

- The aim of this module is to support caregivers in helping their child develop an identity of their choice while providing the freedom to consider potential contributing influences of the family of origin, foster family, and peers. Caregivers are supported in facilitating a multidimensional, nonjudgmental picture of the biological parents that may allow the child to identify with their strengths. Parents are provided with practical assistance on how to reconcile the child’s needs for social belonging and autonomy and how to prevent or reduce potential loyalty conflicts and associated distress across family members.

**Module *My Coach*—providing professional support to the caregiver**

- Professional support is provided to the caregiver in the form of 1 phone call per experimental module and 1 joint call for the basic modules with a coach. Coaching sessions include feedback on the caregivers’ progress in enhancing the elaboration of module content and discussing related issues in the family. The coach answers questions about program content, homework, and the transfer of knowledge and activities in their everyday family life. The participants in conditions with coach assistance *on* receive 50 minutes of additional assistance via phone (per call) as well as either an SMS text message or an email with the core message of the module.

### Data Collection for the Primary and Secondary Outcomes

Each participant will be assessed on the web using *SoSci Survey* [66] 3 times, that is, at preintervention assessment (week 1), postintervention assessment (week 12), and 3-month follow-up assessment (week 24). Families will complete a brief questionnaire (5 minutes) on adverse events and child behavior during the past 2 weeks at the beginning of each module on the program website to ensure a closely monitored adverse event assessment. Multimedia Appendix 5 outlines the SPIRIT-recommended schedule of enrollment, interventions, and assessments. Families will receive a reimbursement of €30 (US $29.32 in the form of 2 vouchers: €20 (US $19.55) for caregivers and €10 (US $9.77) for the child) for their study participation after follow-up assessments.

### Data Management

To ensure data privacy, research data will be identified using pseudonyms and will be stored on 2 password-protected servers only accessible by approved study staff members. Personal data (ie, username, phone number, and email address) will be stored separately from research data. Personal data and the pseudonym codes will only be matched in a handwritten key code list to protect the confidentiality of data. Identifiable data (including the key code list) will be deleted 2 years after the end of the project. The research data will then be anonymized. The anonymized data set will be stored for 10 years and shared with other research teams upon request using a repository that will be chosen by the consortium (advised by the DSMB).

### Data Monitoring

An independent DSMB will provide additional oversight on data safety, ethical procedures, and best clinical practices. A thorough data safety concept was developed and piloted in phase 1. This safety protocol outlines how adverse events will be identified, registered, acknowledged, and handled.

### Measures

#### Primary Measures

To investigate the primary efficacy, we will use a revictimization score derived from 2 measures: the Juvenile Victimization Questionnaire [33] and the bullying screener [34]. We will assess the child and parent reports of each measure while using the parent report for the primary outcome. The Juvenile Victimization Questionnaire is a widely and internationally used self-report measure to assess victimization in children aged 8 to 17 years. It consists of 34 items in the child version and 37 items in the parent version spanning 5 domains, namely, crime, child maltreatment, peer and sibling victimization, sexual victimization, and witnessing crime, with follow-up questions that also assess the frequency and perpetrators of the victimization events. A total of 3 supplemental items on electronic victimization will be assessed. In our study, the participants (caregiver and child) will be asked whether the child was exposed to the respective event and, if yes, whether it happened during the last 3 months (primary outcome–assessment period). The participants will respond with yes (1) or no (0), leading up to a total score, with higher scores indicating greater victimization exposure. The bullying screener [34] is a 6-item screening tool that assesses bullying as victim and offender. After the respective definition of bullying type, the participants are asked how often these things happened to the child or how often they have done this to others in the last 3 months. The participants then respond on a 4-point scale from never to a lot (*at least once a week*).

#### Secondary Outcomes

Secondary outcomes include relationship-related risk-taking behaviors (questionnaire on risky situations in relationships; Heinrichs, N, unpublished data, April 2021). The questionnaire comprises 14 risky situations (eg, “How often has someone done something to your child even though he or she did not want that and said ‘no’?”). Caregivers and children will be asked how often the risky situation occurred during the last 3 months (frequency). Afterward, they will rate how likely it is that the situation is followed by a positive and negative consequence (child’s risk appraisal) on a scale from 1 (very unlikely) to 5 (very likely). The structure (frequency and appraisal of risky situations) is based on the Cognitive Appraisal of Risky Events [67]. In addition, 6 case vignettes for risky situations are currently being developed to administer at postintervention assessment. Risky situations include being persuaded by a friend to swim far out to sea, witnessing bullying behavior in school, or a stranger standing very close to a child at a swimming pool. To assess functional relationship behavior across relationships with caregivers, siblings, and others, we will use 3 well-established measures: (1) the subscales communication and involvement of the Parenting Relationship Questionnaire [35], (2) the 7 subscales referring to “warmth and closeness” of the Sibling Relationship Questionnaires [36-38], and (3) the Relationship Problems Questionnaire [39]. Further details are provided in Multimedia Appendix 2.

#### Mediators and Moderators

Caregivers and children will complete a battery of web-based questionnaires assessing potential moderators and mediators. Multimedia Appendix 2 provides the full list of measures. *Mediators* comprise a range of measures on relationship-related risk-taking cognitions, detection of risk and safety signals in relationships, emotional security, parental discord in front of the child, attachment, caregiver’s support with identity construction, program adherence, self-appraisal, belongingness, emotional regulation, parental self-care, and child behavior problems. Child’s gender, contact with the biological family, type of maltreatment, executive functioning, and parental childhood trauma will be assessed as *moderators*.

### Statistical Analysis

#### Analyses

Before the analyses, missing data will be examined and appropriately handled using multiple imputation or full information maximum likelihood estimation. To investigate the effectiveness of each intervention component, the primary analyses will test the pre–follow-up change in children’s revictimization composite scores. The primary analysis will be conducted in an intention-to-treat sample. We will use the analysis of covariance with main and interaction effects on the primary and secondary end points. The main effects and interactions are estimated based on aggregates (each reflecting the presence or absence of a specific component) across the 16 experimental conditions. The main effects will be modeled as a fixed effect with baseline levels of an outcome as a covariate and an assumed type I error of *P*<.10 (recommended for component selection [22]). To examine the hypotheses related to mediating and moderating effects, we will use regression analyses. Mediational analyses will be conducted by analyzing the indirect effects of each component on the primary outcome via the assumed mediators (Figure 1). Moderator analyses will be modeled in steps with baseline predictors (eg, type of maltreatment) and then as a second model including interactions with the main effect by condition.

#### Decision-making Process

We will use the “all active components criterion” [22] for the selection of component levels. This means that we plan to include the component levels of the entire set of components that are associated with a better outcome, following an a priori decision-making process: (1) main effect on the primary outcome at follow-up; (2) if no significant main effect can be found, we will consider the interaction effect; and (3) if no main or interaction effects are found, we will consider the mediation models. If we do not find any significant main effect for any of the 4 factors on revictimization, we will choose the more cost-effective component levels. Before analysis, the assignment will be blinded in the data set. After completing the decision-making process, the assignment will be unblinded.

## Results

**Figure 3:**
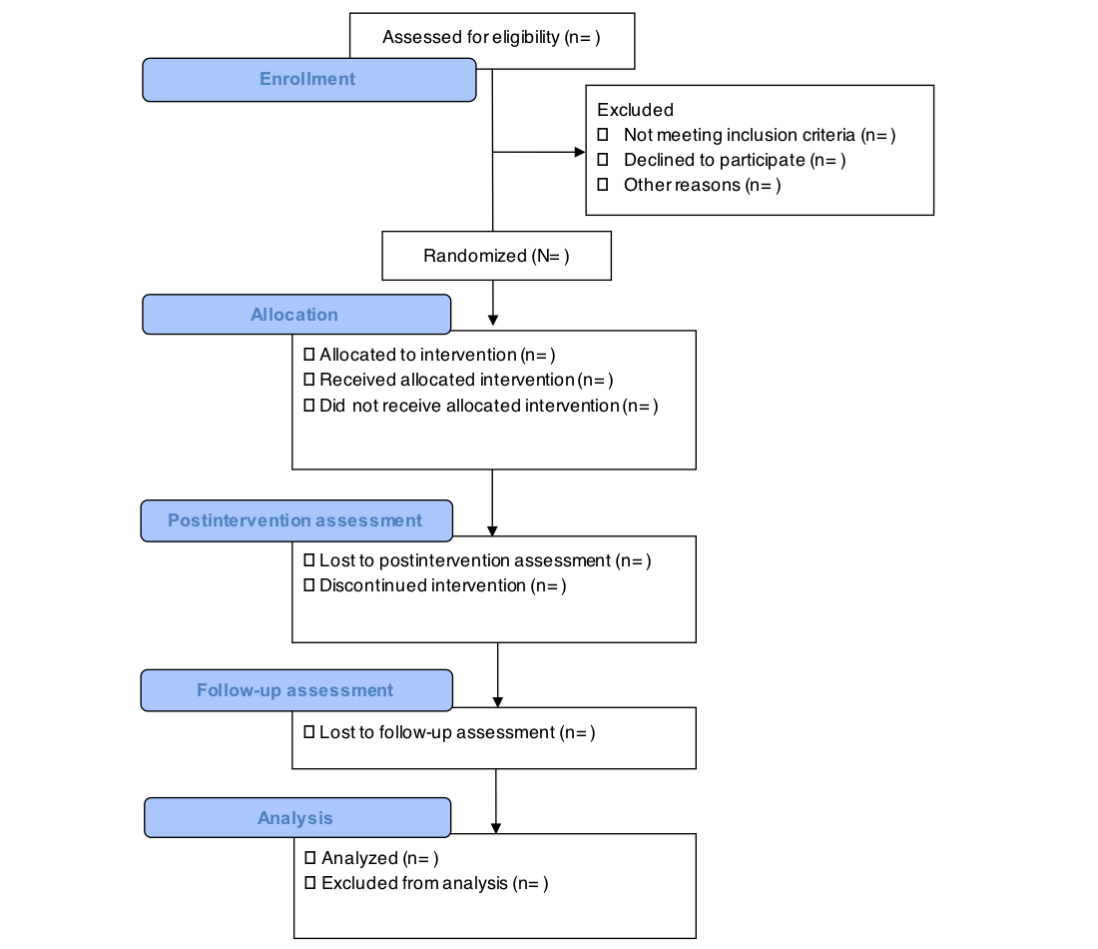
CONSORT (Consolidated Standards of Reporting Trials) flowchart for EMPOWERYOU Subproject 4.

The EMPOWERYOU project was funded in February 2019. Phase 1 (conducting focus groups and developing and piloting the intervention) was completed in September 2021. Afterward, the intervention was adapted based on the results of the pilot study. We started recruitment and data collection for phase 2 (factorial trial) in March 2022. Data collection is ongoing. As of October 2022, we recruited 181 families. Although it is difficult to predict the exact study timeline because of COVID-19 pandemic–related delays, preliminary results are expected in February 2024. Results will be published in peer-reviewed journals and presented at key conferences for researchers and stakeholders. Figure 3 shows the flow diagram of the study.

## Discussion

Caregivers in the foster care system take responsibility for children who are at risk of developing mental health problems because of their early life adversities. Therefore, it is important to provide support in an easy-to-access manner. For this purpose, we developed a consumer-informed e-parenting intervention that is meant to support foster caregivers in their important role of caring for these children. Scalability has been an important factor when developing the intervention (e-intervention will be accessible at any preferred time for caregivers, and costs will be—ignoring the professional support component—primarily driven by website hosting and maintenance), and this factorial trial can make a significant contribution to the optimization of an intervention package, which, after optimization, needs to be tested in a traditional randomized controlled trial (phase 3, evaluation) before being disseminated.

## Appendix

Multimedia Appendix 1 Prior work—development of a conceptual model (phase 1 of the multiphase optimization strategy).

Multimedia Appendix 2 Outcomes and measures.

Multimedia Appendix 3 SPIRIT (Standard Protocol Items: Recommendations for Interventional Trials) checklist for clinical trials.

Multimedia Appendix 4 Informed consent form.

Multimedia Appendix 5 SPIRIT (Standard Protocol Items: Recommendations for Interventional Trials)-recommended schedule of enrollment, interventions, and assessments.

## Abbreviations

DSMB: Data Safety and Monitoring Board
MOST: multiphase optimization strategy
PTSD: posttraumatic stress disorder
SPIRIT: Standard Protocol Items: Recommendations for Interventional Trials

## Data Availability

Data that can be fully anonymized are available from the corresponding author upon reasonable request. Access to data is restricted to the scientific community. The anonymized data set will be stored for 10 years and shared with other research teams upon request using a repository that will be chosen by the consortium (advised by the Data Safety and Monitoring Board, eg, Zenodo).
