# Supplementary material for: Preventing Revictimization Through a Web-Based Intervention for Primary Caregivers of Youth in Care (EMPOWERYOU): Protocol for a Randomized Factorial Trial
Source: JMIR Res Protoc. 2022 Oct 24;11(10):e38183. doi: 10.2196/38183 (PMC9641515; doi:10.2196/38183)
Supplement: Multimedia Appendix 1 [file resprot_v11i10e38183_app1.pdf]

Appendix 1: Prior work – Development of a conceptual model (Phase 1 of MOST)

In order to develop and tailor such services to foster parents and youth in care (Y-IC) with maltreatment experiences in the past, we conducted three focus groups with foster caregivers (*n* = 16), four groups with professionals working in the foster system or care leavers (*n* = 16), one group with foster children (*n* = 4), and three groups with adolescence in care (*n* = 18). Results from the focus groups with adolescence, care leavers, and professionals will be published elsewhere. For the development and selection of the caregiver intervention, we primarily focused on results from the caregiver and child groups (as the primary target group) as well as the professional experts ensuring that there is collaborative and equitable input from all relevant sources of expertise. Focus group discussions were audio recorded, subsequently transcribed and coded using the MAXQDA software [29]. A qualitative content analysis was conducted according to Mayring [30]. Main categories were deductively derived from (re-)victimization theories [27, 28, 31, 32] and included 1) relevant outcomes, 2) risk factors, 3) program content, and 4) didactics reflecting consumer-based needs. Further data driven sub-categories were derived from inductive content analysis. In addition, we reviewed a large number of publications to identify processes that may likely drive the increased risk of revictimization in children with early adverse experiences. Based on the scientific information, we identified several factors that increase the risk for revictimization that likely resulted from the initially adverse experiences in the (biological) maltreating family, at least in part explaining the vicious cycle of victimization and revictimization [31]. Furthermore, we also attended to several theories to further refine our conceptual model [27, 28, 31, 32]. Based on both, the consumer data and the scientific information with a special emphasis on social learning/feminist theory, risk detection and executive functioning theory, we build our conceptual model.

References

27. Ruback RB, Clark VA, Warner C. Why are crime victims at risk of being victimized again? Substance use, depression, and offending as mediators of the victimization-revictimization

link. J Interpers Violence 2014 Jan;29(1):157-185. [doi: 10.1177/0886260513504626] [Medline: 24097905]

28. DePrince AP, Chu AT, Labus J, Shirk SR, Potter C. Testing two approaches to revictimization prevention among adolescent girls in the child welfare system. J Adolesc Health 2015 Feb;56(2 Suppl 2):S33-S39. [doi: 10.1016/j.jadohealth.2014.06.022] [Medline: 25620452]
29. MAXQDA. VERBI Software. Berlin, Germany: VERBI Software; 2019. URL: https://www.maxqda.com/about [accessed 2021-07-01]
30. Mayring P. Qualitative Inhaltsanalyse: Grundlagen und Techniken. 12th Auflage. Weinheim, Germany: Julius Beltz; 2010.
31. Castro A, Ibáñez J, Maté B, Esteban J, Barrada JR. Childhood sexual abuse, sexual behavior, and revictimization in adolescence and youth: a mini review. Front Psychol 2019 Aug 30;10:2018 [FREE Full text] [doi: 10.3389/fpsyg.2019.02018] [Medline: 31543854]
32. Fraley RC, Roisman GI. Do early caregiving experiences leave an enduring or transient mark on developmental adaptation? Curr Opin Psychol 2015 Feb;1:101-106. [doi: 10.1016/j.copsyc.2014.11.007]
