# Supplementary material for: Preventing Revictimization Through a Web-Based Intervention for Primary Caregivers of Youth in Care (EMPOWERYOU): Protocol for a Randomized Factorial Trial
Source: JMIR Res Protoc. 2022 Oct 24;11(10):e38183. doi: 10.2196/38183 (PMC9641515; doi:10.2196/38183)
Supplement: Multimedia Appendix 2 [file resprot_v11i10e38183_app2.pdf]

Appendix 2: Outcomes and measures

Caregivers and/or children will complete the following online questionnaires at pre-assessments, post-assessments and/or 3-months (3M) follow-up-assessments. All measures were translated into German language (if necessary).

Table A1. Outcomes and measures.

Variables	Measures	Items	Assess-ment	Source
**Demographics**				
Demographic characteristics on child and caregiver	Demographics questionnaire [40]	30-39 (depends on filter)	Pre	Caregiver
**Primary Outcomes**				
Victimization in form of child maltreatment, neglect and bullying by peers and siblings	The Juvenile Victimization Questionnaire, JVQ, [33]	40-79 (parent), 37-74 (child)	Pre, 3M	Caregiver and child
Victimization in form of cyber bullying	Bullying Screener [34]	12 (parent), 6 (child)	Pre, 3M	Caregiver and child
**Proximal Outcomes**				
Relationship-related risk-taking behaviors	Questionnaire for risky situations in relationships, frequencies [Heinrichs, N, unpublished measure, April 2021]	14 (parent), 14 (child)	Pre, post and 3M	Caregiver and child
	Risk-Check Vignettes [Niestroj, S, unpublished measure, September 2021]	6 (parent), 6 (child)	Post	Caregiver and child
Functional relationship behavior (in relationships with caregiver, siblings, peers, and unspecified)	Parenting Relationship Questionnaire, PRQ, subscales: communication, involvement, version ages 6–18 [35]	22	Pre, post and 3M	Caregiver
	Sibling Relationship Questionnaires, SRQ-deu, subscales on warmth and closeness [36-38]	21 (parent), 29 (child)	Pre, post and 3M	Caregiver and child
	Relationship Problems Questionnaire, RPQ [39, 41]	10	Pre, post and 3M	Caregiver
**Mediators**				
Relationship-related risk-taking cognitions	Questionnaire for risky situations in relationships, subscale: evaluation [Heinrichs, N, unpublished measure, April 2021]	28 (parent), 28 (child)	Pre, post and 3M	Caregiver and child

	Risk-Check Vignettes, subscale: cognitions [Niestroj, S, unpublished measure, September 2021]	18 (parent), 18 (child)	Post	Caregiver and child
Detection of risk signals in relationships	Inventory for recording impulsiveness, risky behavior and empathy in children aged 9 to 14, IVE, subscales: impulsiveness, risky behavior, empathy [42]	48	Pre, post and 33M	Child
	Risk-Check Vignettes, subscale: emotions [Niestroj, S, unpublished measure, September 2021]	18 (parent), 18 (child)	Post	Caregiver and child
Detection of safe signals in relationships	Network of Relationships Inventory: Behavioral Systems Version, subscales: seeks & provides safe haven, seeks & provides base, companionship [43]	15 (parent), 16 (child)	Pre, post and 3M	Caregiver and child
Emotional security	Security in the Interparental Subsystem Scale— Child Report, SIS; subscales: emotional reactivity, behavioral dysregulation, avoidance, involvement, constructive family representations, destructive family representations [44, Translation: Zemp, M, unpublished measure]	31 (if in relationship)	Pre, post and 3M	Caregiver and child
Parental discord in front of the child	O'Leary-Porter Scale [45]	10 (if in relationship)	Pre, post and 3M	Caregiver
Attachment	Parenting Relationship Questionnaire, subscale attachment [35]	15	Pre, post and 3M	Caregiver
Caregiver´s support with identity constructing	Questionnaire of parental support for child identity development [Brühl, A, unpublished measure, April 2021]	27	Pre, post and 3M	Caregiver
Self-appraisal	Items on "self-concept" derived from the The National Longitudinal Study of Adolescent to Adult Health, Add Health [46]	7	Pre, post and 3M	Caregiver and child
Belongingness (family and peers at school)	Items on "parent–family connectedness" and "school-belonging" derived from the The National Longitudinal Study of	11	Pre, post and 3M	Caregiver and child

	Adolescent to Adult Health, Add Health [46-50]			
Program adherence/engagement	EMPOWERYOU-program content questionnaire [Heinrichs, N, unpublished measure, August 2020]	6-47 (depends on allocation)	Post	Caregiver
	Self-made checklist for completed items/tasks in each module	50	After 4 weeks and post	Caregiver
**Moderators**				
Gender and contact with biological family	Demographics questionnaire [40]	Covered above	Pre	Caregiver
Type of maltreatment (i.e., physical abuse, sexual abuse, emotional abuse, neglect)	The Juvenile Victimization Questionnaire, JVQ, lifetime prevalence [33]	Covered above	Pre and 3M	Caregiver
Executive functioning	Short version of the BRIEF - Behavior Rating Inventory of Executive Function 6-16 J. [51]	12	Pre	Caregiver
Parental Childhood Trauma	Childhood Trauma Questionnaire, CTQ [52]	28	Pre	Caregiver
**Mediators (not included in model)**				
Self-worth	Items on "self-concept" derived from the The National Longitudinal Study of Adolescent to Adult Health, Add Health [46]	Covered above	Pre, post and 3M	Caregiver and child
Parental self-care	Parenting Relationship Questionnaire, subscale relational frustration [35]	16	Pre, post and 3M	Caregiver
Emotional regulation	FEEL – KJ short version [53]	30	Pre, post and 3M	Caregiver
Internal and external behavior problems	The Strengths and Difficulties Questionnaire, SDQ-Deu [54]	25	Pre, post and 3M	Caregiver
**Screening**				
Acute child endangerment	Self-made items	5	Pre	Caregiver
**Biweekly change**				
Parent reporting scale	Parent Daily Report, Fast track version [55]	4	Biweekly	Caregiver
Adverse Events	Adverse Event Monitoring Checklist [Heinrichs, N, unpublished measure, March 2022]	21	Biweekly	Caregiver

References

33. Finkelhor D, Ormrod R, Turner H, Hamby SL. The victimization of children and youth: a comprehensive, national survey. Child Maltreat 2005 Feb;10(1):5-25. [doi: 10.1177/1077559504271287] [Medline: 15611323]
34. Wolke D, Lereya ST. Long-term effects of bullying. Arch Dis Child 2015 Sep;100(9):879-885 [FREE Full text] [doi: 10.1136/archdischild-2014-306667] [Medline: 25670406]
35. Kamphaus RW, Reynolds CR. Parenting Relationship Questionnaire (PRQ). London, UK: Pearson; 2006.
36. Furman W, Buhrmester D. Children's perceptions of the qualities of sibling relationships. Child Dev 1985 Apr;56(2):448-461. [doi: 10.2307/1129733]
37. Bojanowski S, Riestock N, Nisslein J, Weschenfelder-Stachwitz H, Lehmkuhl U. Psychometrische Gütekriterien der deutschen Version des Sibling Relationship Questionnaire (SRQ-deu). Psychother Psychosom Med Psychol 2015 Sep;65(9-10):370-378. [doi: 10.1055/s-0035-1547228] [Medline: 26039367]
38. Bojanowski S, Riestock N, Nisslein J. Deutsche Version des Sibling Relationsship Questionnaire (SRQ-deu): Normierung an einer Schülerstichprobe. Psychother Psychosom Med Psychol 2019 Feb;69(2):81-86. [doi: 10.1055/a-0574-2306] [Medline: 29660750]
39. Minnis H, Rabe-Hesketh S, Wolkind S. Development of a brief, clinically relevant, scale for measuring attachment disorders. Int J Methods Psychiatr Res 2002;11(2):90-98 [FREE Full text] [doi: 10.1002/mpr.127] [Medline: 12459799]
40. Chodura S, Lohaus A, Symanzik T, Möller C, Heinrichs N, Konrad K. Demografische Eigenschaften von Pflegefamilien in Deutschland. Z Kinder Jugendpsychiatr

Psychother 2019 May;47(3):211-227. [doi: 10.1024/1422-4917/a000620] [Medline: 30264651]

41. Schröder M, Fux E, Lüdtke J, Izat Y, Bolten M, Schmid M. German version of the relationship problems questionnaire: effective screening for attachment disorder. Psychopathology 2019;52(6):334-345. [doi: 10.1159/000504675] [Medline: 31865355]
42. von Stadler C, Janke W, Schmeck K. Inventar zur Erfassung von Impulsivität, Risikoverhalten und Empathie bei 9- bis 14-jährigen Kindern. Göttingen, Germany: Hogrefe Verlag; 2004.
43. Furman W, Buhrmester D. The network of relationships inventory: behavioral systems version. Int J Behav Dev 2009 Sep 01;33(5):470-478 [FREE Full text] [doi: 10.1177/0165025409342634] [Medline: 20186262]
44. Davies PT, Forman EM, Rasi JA, Stevens KI. Assessing children's emotional security in the interparental relationship: the Security in the Interparental Subsystem Scales. Child Dev 2002;73(2):544-562. [doi: 10.1111/1467-8624.00423] [Medline:11949908]
45. Porter B, O'Leary KD. Marital discord and childhood behavior problems. J Abnorm Child Psychol 1980 Sep;8(3):287-295. [doi: 10.1007/BF00916376] [Medline: 7410730]
46. Mueller CE. Protective factors as barriers to depression in gifted and nongifted adolescents. Gifted Child Q 2009 Jan 01;53(1):3-14. [doi: 10.1177/0016986208326552]
47. Jacobson KC, Rowe DC. Genetic and environmental influences on the relationships between family connectedness, school connectedness, and adolescent depressed mood: sex differences. Dev Psychol 1999 Jul;35(4):926-939. [doi: 10.1037//0012-1649.35.4.926] [Medline: 10442862]

48. Resnick MD, Bearman PS, Blum RW, Bauman KE, Harris KM, Jones J, et al. Protecting adolescents from harm. Findings from the National Longitudinal Study on Adolescent Health. JAMA 1997 Sep 10;278(10):823-832. [doi: 10.1001/jama.278.10.823] [Medline: 9293990]

49. Anderman LH. Academic and social perceptions as predictors of change in middle school students' sense of school belonging. J Exp Educ 2003 Jan;72(1):5-22. [doi: 10.1080/00220970309600877]

50. Anderman EM. School effects on psychological outcomes during adolescence. J Educ Psychol 2002 Dec;94(4):795-809. [doi: 10.1037/0022-0663.94.4.795]

51. Drechsler R, Steinhausen HC. Verhaltensinventar zur Beurteilung exekutiver Funktionen: Deutschsprachige Adaptation des Behavior Rating Inventory of Executive Function (BRIEF). Göttingen, Germany: Hogrefe Publishing; 2013.

52. Bernstein DP, Fink L, Handelsman L, Foote J, Lovejoy M, Wenzel K, et al. Initial reliability and validity of a new retrospective measure of child abuse and neglect?: reply. Am J Psychiatry 1995 Oct;152(10):1535-1537. [doi: 10.1176/ajp.152.10.1535-a]

53. Greuel JF, Briegel W, Heinrichs N. Die Eltern-Kurzversion des Fragebogens zur Erhebung der Emotionsregulation bei Kindern und Jugendlichen (FEEL-KJ). Z Klin Psychol Psychother 2018 Jul 10;47(1):48-58. [doi: 10.1026/1616-3443/a000463]

54. Goodman A, Lamping DL, Ploubidis GB. When to use broader internalising and externalising subscales instead of the hypothesised five subscales on the Strengths and Difficulties Questionnaire (SDQ): data from British parents, teachers and children. J Abnorm Child Psychol 2010 Nov;38(8):1179-1191. [doi: 10.1007/s10802-010-9434-x] [Medline: 20623175]

55. Hurley SM. Parent Daily Report - Child Behavior Items. Fast Track Project Technical Report. 2000 Oct. URL: https:/ /fasttrackproject.org/wp-content/uploads/sites/31/2021/07/pdr5tech.pdf [accessed 2022-10-13]
