# Supplementary material for: Preventing Revictimization Through a Web-Based Intervention for Primary Caregivers of Youth in Care (EMPOWERYOU): Protocol for a Randomized Factorial Trial
Source: JMIR Res Protoc. 2022 Oct 24;11(10):e38183. doi: 10.2196/38183 (PMC9641515; doi:10.2196/38183)
Supplement: Multimedia Appendix 4 [file resprot_v11i10e38183_app4.pdf]

Appendix 4: Informed consent (in German language)

Informationen zum Forschungsvorhaben

Liebe Familien,
wir freuen uns über Ihr Interesse an unserem Vorhaben!

Welche Ziele verfolgen wir?
Im Rahmen dieser Studie **EMPOWERYOU** werden die Inhalte (="Module") eines neu entwickelten Online-Programmes für Pflege- und Adoptiveltern mit Kindern im Alter von 8 bis 13 Jahren auf ihre jeweilige Wirksamkeit hin überprüft. Das Ziel dieses interaktiven Online-Programmes ist, Eltern im Umgang mit den Bedürfnissen ihres aufgenommenen Kindes zu unterstützen und Eltern dabei zu helfen, ihr Kind in Pflege vor (erneuten) Gewalterfahrungen zu schützen. Wenn das Programm Familien hilft, werden wir es nach der Studie veröffentlichen, damit alle Pflege- und Adoptivfamilien in Deutschland das Programm kostenfrei nutzen können.

In dem Programm erwarten Sie spannende Informationen, Hörspiele, Videos und Übungen. Dies wird etwa 45 Minuten pro Woche in Anspruch nehmen. Zudem erhalten Sie konkrete Empfehlungen und Übungen, die Sie gemeinsam mit Ihrem Kind in Ihrem Alltag ausprobieren können.
Sie werden lernen, …

- wie Sie als Eltern Ihre eigenen Bedürfnisse erkennen und was Sie brauchen, um selber stark sein zu können
- wie Sie den Selbstwert Ihres Kindes fördern
- wie Sie Ihre Gefühle und die Ihres Kindes erkennen und mit ihnen umgehen können
- wie Ihr Kind Warnsignale und Gefahren in Beziehungen erkennt und sich selbstsicher verhält
- wie Ihr Kind sich in Beziehungen zu Erwachsenen und Gleichaltrigen sicher fühlen kann
- wie Sie die Identitätsentwicklung Ihres Kindes fördern können

Was kommt auf Sie zu?
Während Sie sich auf der Webseite www.empoweryou-programm.de registrieren, können Sie zusammen mit diesem Informationsschreiben eine Einwilligungserklärung online einsehen. Bitte lesen Sie beide Dokumente aufmerksam durch. Sollten Sie dazu Fragen haben, können Sie uns gerne anrufen (M.Sc. Antonia Brühl, Tel.: +49 421 - 218 68542). Wenn Sie sich dazu entscheiden, an der Studie teilzunehmen, klicken Sie bitte anschließend auf „Ich bin damit einverstanden, an EMPOWERYOU teilzunehmen und willige in die entsprechende Verarbeitung meiner Daten ein.“ Wenn zudem Sie und Ihr Kind in Pflege damit einverstanden sind, dass Ihr Kind an der Studie teilnimmt (indem es drei Online-Fragebögen ausfüllt), klicken Sie bitte anschließend auf „Mein Kind in Pflege und ich sind damit einverstanden, dass mein Kind an EMPOWERYOU teilnimmt und wir willigen in die entsprechende Verarbeitung seiner/ihrer Daten ein.“ Sobald Sie der Einverständniserklärung zugestimmt und den Registrierungsprozess abgeschlossen haben, erhalten Sie in den nächsten Tagen eine E-Mail mit einem Link zu der ersten Online-Befragung (s. unten Online-Fragebögen). Anschließend erhalten Sie Zugang zu dem internen Bereich und den Modulen des Onlineprogrammes. Das gesamte Programm geht über 10 Wochen und besteht aus 5 Modulen zu unterschiedlichen Themen. Sie können alle diese Module kostenfrei nutzen. Wenn Sie sich für die Studie anmelden, wird per Zufall entschieden, welche Module Sie direkt nutzen können und welche Module erst nach 24 Wochen für Sie freigeschaltet werden. Das ist notwendig, damit wir überprüfen können, welche Module besonders gut helfen.

Professionelle Unterstützung durch einen Coach
Zudem entscheidet der Zufall, ob Sie zusätzlich zu den Modulen Telefonate mit einem*r Psychotherapeut*in in Anspruch nehmen können. Diese*r wird Eltern bei der Durchführung des Programmes unterstützen und beratend zur Seite stehen. Da auch hier geprüft werden soll, ob oder unter welchen Bedingungen eine solche zusätzliche Unterstützung für Sie als Eltern mit Adoptiv- oder Pflegeaufgaben nützlich ist, werden auch hierfür die Teilnehmenden per Zufall ausgewählt.

Online-Fragebögen
Um zu überprüfen, ob Ihnen das Online-Programm hilft, bitten wir Sie und Ihr Kind in Pflege vor dem Programm, nach dem Programm (ca. nach 10 Wochen) und 3 Monate nach Programmende einen Online-Fragebogen auszufüllen. Hierfür erhalten Sie separate Links in einer E-Mail. Die Fragen beziehen sich u.a. auf das Verhalten Ihres Kindes, dessen Beziehungen und mögliche Konflikte zu Gleichaltrigen und Geschwistern, sowie um Ihre eigenen Belastungen, denen Sie als Elternteil ausgesetzt sein können. Im Rahmen von der Untersuchung von Präventionsprogrammen ist es zudem wichtig, die körperliche und seelischen Gesundheit während der Studienteilnahme zu untersuchen. Aus diesem Grund werden wir Ihnen auch Fragen zu Ihrer körperlichen und seelischen Gesundheit sowie der Ihres Kindes stellen. Hierbei handelt es sich um Gesundheitsdaten als personenbezogene Daten besonderer Kategorien (Art. 9 DSGVO). Das Ausfüllen der Eltern-Fragebögen wird ungefähr 90 Minuten dauern. Das Ausfüllen der Fragebögen für Ihr Kind Pflege dauert etwa 30 Minuten. Zusätzlich werden nur Sie als Eltern nach jedem Modul (alle zwei Wochen) einen kurzen Online-Fragebogen ausfüllen, der etwa 5 Minuten dauert.

Datenschutz
Der Umgang mit Ihren Daten und den Untersuchungsergebnissen entspricht den Vorgaben der EU-Datenschutzgrundverordnung (DSGVO). Sämtliche personenbezogene Informationen (Ihr Benutzername, E-Mail-Adresse und Telefonnummer), die wir im Rahmen des Projektes von Ihnen erhalten, werden absolut vertraulich behandelt. Alle Mitarbeiter*innen des Projektes unterliegen der Schweigepflicht und dürfen keine Informationen an Dritte weitergeben. Im Fall einer bekanntwerdenden Kindeswohlgefährdung müssen wir jedoch das Jugendamt informieren. Dieses Vorgehen würden wir zuvor mit Ihnen besprechen.

Die Erhebung und Verarbeitung persönlicher Daten erfolgen pseudonymisiert an der Universität Bremen, Institut der Psychologie – Klinische Psychologie und Psychotherapie unter Verwendung einer Nummer und ohne Angabe Ihres Namens. Es existiert eine Kodierliste auf Papier, die Ihren Benutzernamen sowie Ihre Kontaktdaten (Telefonnummer, Emailadresse) mit der Nummer verbindet. Die Kodierliste ist nur den Wissenschaftler*innen in diesem Projekt am Standort Bremen zugänglich; das heißt, nur diese Personen können die erhobenen Daten mit Ihrem Namen und Ihren Kontaktdaten in Verbindung bringen. Ausschließlich pseudonymisierte Forschungsdaten werden auch Mitarbeitenden im **EMPOWERYOU** Konsortium an den Standorten Aachen, Berlin, Bielefeld und Karlsruhe zur Verfügung gestellt. Die pseudonymisierten Daten werden ohne Namensnennung an die folgenden vier Forschungsgruppen im EMPOWERYOU Konsortium weitergegeben:

- Prof. Dr. Kerstin Konrad
  Uniklinik Aachen, Lehr- und Forschungsgebiet für klinische Neuropsychologie des Kindes- und Jugendalters
  Neuenhofer Weg 21, 52074 Aachen
  und deren Stellvertreter*innen
- Prof. Dr. Arnold Lohaus
  Universität Bielefeld, Fakultät für Psychologie und Sportwissenschaft, Abteilung für Psychologie
  Postfach 100131, 33501 Bielefeld
  und dessen Stellvertreter*innen
- Prof. Dr. Ulrich Ebner-Priemer
  KIT, Institut für Sport und Sportwissenschaft
  Engler-Bunte-Ring 15, 76131 Karlsruhe
  und dessen Stellvertreter*innen
- Prof. Dr. Birgit Wagner
  Medical School Berlin, Klinische Psychologie & Psychotherapie – Verhaltenspsychologie
  Calandrellistraße 1-9, 12247 Berlin
  und deren Stellvertreter*innen

Die Kodierliste mit Ihren personenbezogenen Informationen wird ausschließlich am Standort Bremen in einem abschließbaren Schrank aufbewahrt und nach Abschluss des Projektes vernichtet (spätestens 2 Jahre nach Projektende). Ihre Daten sind dann anonymisiert. Damit ist es niemandem mehr möglich, die erhobenen Daten mit Ihrem Namen und Ihren Kontaktdaten in Verbindung zu bringen. Die anonymisierten Daten werden 10 Jahre gespeichert. Solange die Kodierliste existiert, können Sie die Löschung aller von Ihnen erhobenen Daten verlangen. Ist die Kodierliste aber erst einmal gelöscht, können wir Ihren Datensatz nicht mehr identifizieren. Deshalb können wir Ihrem Verlangen nach Löschung Ihrer Daten nur solange nachkommen, wie die Kodierliste existiert. Die vollständig anonymisierten Daten sind auf Anfrage anderen Forschungsgruppen online zugänglich. Dieses Vorgehen dient der Sicherstellung guter wissenschaftlicher Arbeit. Andere Forschende können dadurch beispielsweise die Auswertung nachvollziehen oder eine alternative Auswertung testen. Eine Zuordnung der Daten zu Ihrer Person ist nicht möglich. Bei Interesse werden wir Sie nach Abschluss des Forschungsprojekts gerne über die Ergebnisse informieren.

Was sind Risiken Ihrer Teilnahme an der Studie?
Die Online-Befragung sowie das Online-Programm können potentiell belastende Themen (z.B. Mobbingerfahrungen der Kinder) beinhalten. Diese werden aber nicht über Ihre Alltagserfahrungen im Zusammenleben mit dem aufgenommenen Kind hinaus gehen.

Vergütung
Für die Teilnahme an der Studie **EMPOWERYOU** erhält Ihre Familie eine Aufwandsentschädigung in Form von zwei Gutscheinen in Höhe von 20€ (für Sie) und 10€ (für Ihr Kind). Die Gutscheine werden Ihnen per E-Mail zugesendet.

Freiwilligkeit
Die Teilnahme an dem Forschungsprojekt ist freiwillig. Sie und Ihr Kind können Ihre Einwilligung zur Teilnahme jederzeit und ohne Angabe von Gründen widerrufen. Durch ein Widerrufen entstehen Ihnen in keiner Form irgendwelche Nachteile.

Wir würden uns sehr freuen, wenn Sie sich bereit erklären, uns bei unserem Vorhaben zu unterstützen. Für Fragen stehen wir Ihnen jederzeit gerne zur Verfügung (Ansprechpartnerin: M.Sc. Antonia Brühl, Telefon: +49 421 - 218 68542).

Vielen Dank und herzliche Grüße
Ihr Studienteam

<u>Einwilligungserklärung</u>

Ich habe die Informationen zum Forschungsvorhaben EMPOWERYOU gelesen und den Inhalt verstanden. Mir ist bekannt, dass ich im Rahmen dieser Untersuchung an einem kostenlosen Online-Programm für Pflege- und Adoptiveltern teilnehmen werde und Informationen über meine Erfahrungen als Elternteil und über mein Kind in Pflege gesammelt werden. Ich bin damit einverstanden, dass diese in der Online-Befragung von mir und meinem Kind in Pflege/Adoption preisgegebenen Informationen der „Universität Bremen, Institut der Psychologie – Klinische Psychologie und Psychotherapie“ zur Auswertung zur Verfügung gestellt werden.

Ich habe die Ziele und die Vorgehensweise der Untersuchung verstanden. Ich bin damit einverstanden, dass Gesundheitsdaten als personenbezogene Daten besonderer Kategorien (Art. 9 DSGVO) von mir und meinem Kind in Pflege erhoben werden. Ich weiß, dass mein Kind und ich die Möglichkeit haben, jederzeit Fragen zu stellen. Mein Kind und ich nehmen freiwillig an diesen Untersuchungen teil. Die Speicherung und Auswertung der erfragten Daten erfolgen pseudonymisiert an der Universität Bremen, unter Verwendung einer Nummer und ohne Angabe meines Namens. Es existiert eine Kodierliste auf Papier, die meinen selbstgewählten Benutzernamen und meine E-Mailadresse mit dieser Nummer verbindet. Diese Kodierliste ist nur den Versuchsleiter*innen und der Projektleitung zugänglich, das heißt, nur diese Personen können die erhobenen Daten mit meinem Benutzernamen in Verbindung bringen. Ich erkläre mich damit einverstanden, dass im Rahmen von EMPOWERYOU

pseudonymisierte Daten (ohne Namensnennung) an die Mitarbeitenden im EMPOWERYOU Konsortium an den Standorten Aachen, Berlin, Bielefeld und Karlsruhe weitergegeben werden. Die personenbezogenen Daten werden Dritten außerhalb des Forschungsprojektes am Standort Bremen nicht zugänglich gemacht. Nach Abschluss des Projektes wird die Kodierliste gelöscht. Mir ist bekannt, dass ich mein Einverständnis zur Aufbewahrung bzw. Speicherung dieser Daten widerrufen kann ohne, dass mir daraus Nachteile entstehen. Ich bin darüber informiert worden, dass ich eine Löschung all meiner Daten verlangen kann, solange die Kodierliste noch nicht vernichtet wurde (spätestens 2 Jahre nach Projektende). Nach der Vernichtung der Kodierliste sind unsere Daten anonymisiert. Die wissenschaftliche Auswertung der Untersuchungsergebnisse erfolgt ausschließlich in anonymisierter und zusammenfassender Form. Rückschlüsse auf Einzelpersonen sind nicht möglich.

Mir wurde versichert, dass der Umgang mit meinen Daten und den Untersuchungsergebnissen den Vorgaben der Europäischen Datenschutzverordnung (DSGVO) entspricht.

Ich bin damit einverstanden, an **EMPOWERYOU** teilzunehmen und willige in die entsprechende Verarbeitung meiner Daten ein.
Ja O Nein O

Mein Kind in Pflege und ich sind damit einverstanden, dass mein Kind an **EMPOWERYOU** teilnimmt und wir willigen in die entsprechende Verarbeitung seiner/ihrer Daten ein.
Ja O Nein O
