# Supplementary material for: Preventing Revictimization Through a Web-Based Intervention for Primary Caregivers of Youth in Care (EMPOWERYOU): Protocol for a Randomized Factorial Trial
Source: JMIR Res Protoc. 2022 Oct 24;11(10):e38183. doi: 10.2196/38183 (PMC9641515; doi:10.2196/38183)
Supplement: Multimedia Appendix 5 [file resprot_v11i10e38183_app5.pdf]

Appendix 5: SPIRIT (Standard Protocol Items: Recommendations for Interventional Trials)-recommended schedule of enrollment, interventions, and assessments

Table A3. SPIRIT schedule of enrollment, interventions, and assessments.

		**Study period**										
		Enrolment		Allocation	Post-allocation							
					Intervention period							
	Timepoint		Pre[a]		M1[b]	M2[c]	M3[d]	M4[e]	M5[f]	M6[g]	post[h]	FU[i]
**Enrolment**												
	Eligibility screen	x										
	Informed consent	x										
	Randomization			x								
**Intervention**					x	x	x[j]	x[j]	x[j]	x[j]		
**Assessments**												
	Socio-demographics		x									
	Primary outcomes		x									x
	Secondary outcomes		x								x	x
	Moderators		x									
	Mediators		x			x[k]					x	x
	(S)AE Management		x		x	x	x	x	x		x	x
	Intervention related measures				x	x	x[j]	x[j]	x[j]			

[a]Pre-assessment at week 1

[b]Module 1 at week 2-3 (Parental Self-Care)

[c]Module 2 at week 4-5 (Emotion Regulation)

[d]Module 3 at week 6-7 is component 1 (Relationship-related safety)

[e]Module 4 at week 8-9 is component 2 (Relationship-related risk)

[f]Module 5 at week 10-11 is component 3 (Identity)

[g]Module 6 is component 4 (Coach across module 2, 3, 4 and 5)

[h]Post-assessment at week 12

[i]3-months follow-up assessment at week 24

[j]If randomized

[k]One measure

[l]Severe adverse events
